# Using Photovoice to explore adults’ perceptions towards obesity and factors influencing food choice and physical activity in North Lebanon

**DOI:** 10.1017/S1368980025101328

**Published:** 2025-10-13

**Authors:** Malika Al Kattan, Stephen Fallows, Lynne Kennedy

**Affiliations:** 1 University of Westminster, College of Liberal Arts and Sciences, School of Life Sciences, 115 New Cavendish Steet, London W1W 6UW, UK; 2 University of Chester, Division of Allied Sciences, School of Allied and Public Health, Parkgate Road, Chester CH1 4BJ, UK; 3 Department of Health Sciences, Zayed University, Abu Dhabi, FF1-1-60, United Arab Emirates

**Keywords:** Obesity determinants, Social-ecological model, Photovoice, Food choice, Qualitative research

## Abstract

**Objective:**

To explore adults’ perceptions towards obesity and factors influencing eating behaviour and physical activity in North Lebanon, using a variation of the Photovoice method.

**Design:**

This research is part of a broader qualitative study exploring factors influencing the rising levels of obesity and understanding the barriers and enablers for effective policy for obesity prevention using a socio-ecological model as a guiding framework. For this study, a variation of ‘Photovoice’ was used to collect photographs to explore participants’ perspectives of obesity and its causes in Lebanon, using these photographs to generate discussion in one-to-one face-to-face interviews. Inductive and deductive thematic analyses were used to analyse the transcribed interviews.

**Setting:**

Tripoli, North Governorate, Lebanon.

**Participants:**

Twenty Lebanese adults aged 20–64 years were recruited.

**Results:**

The participants (*n* 20) generated 257 photographs representing both positive and negative influences related to food choice and physical activity, and the various factors perceived to be associated with rising obesity in Lebanon: changes in the food and eating landscape, sedentary behaviours, food environments, eating out and food marketing on social media platforms. Several themes specific to Lebanon were also identified, including the perceptions towards obesity, the central role of women in Lebanese food preparation and the family and the sociocultural importance of food and social gatherings.

**Conclusions:**

This study highlights how influences across the five levels of the socio-ecological model shaped the participants’ food choices and physical activity levels. Collaborative initiatives and public policies are necessary to address the identified barriers and curb the increasing prevalence of obesity in Lebanon.

The traditional Lebanese diet is a variation of the Mediterranean diet, widely recognised as associated with significant health benefits^(1–3)^. Since the late twentieth century, Lebanon’s economy has faced significant instability, and the Lebanese population has undergone a nutrition transition, shifting away from its traditional Mediterranean diet towards Western-style foods and patterns of eating^(4,5)^. The population has also become increasingly sedentary. According to the WHO, nearly half (40 %) of adult men and a third (33 %) of women in Lebanon were physically inactive in 2018^(6)^. A sharp rise in obesity prevalence has mirrored these lifestyle changes in the country. Between 1997 and 2009, obesity prevalence increased among men from 14 to 26·4 % and from 19 to 25·9 % in women^(7)^. Moreover, Lebanese men’s and women’s mean BMI increased substantially more than the estimated international increase: 1·84 kg/m^2^ *v*. 0·4 kg/m^2^ per decade for men and 1·36 kg/m^2^ *v*. 0·5 kg/m^2^ per decade for women^(7)^. Similar trends have been observed in other Arab countries, in the Middle East and North Africa region, where a nutrition transition away from traditional diets has been accompanied by a rise in obesity prevalence^(8)^.

These shifts in dietary patterns and physical activity, alongside the increasing prevalence of obesity in Lebanon, highlight the importance of exploring their underlying causes. Traditionally, obesity has been investigated through a biomedical lens, which identifies obesity as a result of a ‘*positive energy balance*’ caused by excessive caloric intake and insufficient energy expenditure^(9)^. As a result, individual choice and responsible lifestyle choices have been the focus of obesity prevention. However, critics argue that the biomedical view is an ‘oversimplification’ of the mechanism causing obesity^(10)^ and ignores the broader social and environmental contexts of increasingly obesogenic environments^(11)^. Effective prevention, therefore, requires a broader, socio-ecological approach, which considers the complexity of human behaviours and the wider environment (e.g. access to and affordability of healthy lifestyle choices) and social determinants of health (e.g. poverty, social status)^(12,13)^. The socio-ecological model highlights the interplay between influencing factors at multiple levels of influence: intrapersonal, interpersonal, institutional/organisational, community and public policy^(14)^. Thus, the interplay of influences at each level needs to be explored to understand what influences health behaviours and outcomes.

In Lebanon, published studies on obesity have primarily used cross-sectional designs and quantitative methods to examine the correlation of dietary habits, physical activity and socio-economic factors with obesity^(5,15,16)^. While these studies are important to understand associations between different variables (e.g. socio-economic status, education, dietary intake and BMI), these studies offer limited insight into the broader sociocultural and contextual factors that shape Lebanese individuals’ experiences and influence their dietary behaviours and physical activity. To date, only one qualitative study has utilised a socio-ecological model to explore the factors influencing obesity, but it focused exclusively on adolescents^(17)^, and there remains a lack of research exploring Lebanese adults’ perspectives using this framework. This study addresses this gap by qualitatively exploring the perspectives of Lebanese adults living in the North Governorate. It aims to understand their perceptions towards obesity, the socio-ecological factors shaping their food choices and physical activity levels and to inform future public health policies and actions to prevent obesity.

## Methods

This research is part of a broader qualitative study exploring factors influencing the rising levels of obesity and understanding the barriers and enablers for effective policy for obesity prevention using the socio-ecological model^(14)^. For this sub-study, an adapted version of Photovoice was used to guide data collection, where the process was informed by phenomenological inquiry to encourage discussion of ‘*lived experiences*’ and focus on the narratives generated by the photographs collected^(18)^. This version was adopted to help develop a socially situated understanding of the growing prevalence of obesity in Lebanon. Photovoice is a qualitative method where participants take photographs or digital images of people, objects, physical structures or processes resonating with their perception of the phenomenon of interest^(19,20)^. These photographs are subsequently used to facilitate discussion through face-to-face interviews^(19)^. For the interviews, we developed a flexible semi-structured guide (Box 1) to discuss the photographs taken by the participants and encourage culturally sensitive conversations about the rising levels of obesity in Lebanon and possible explanations. The principal author, MAK, undertook the fieldwork as part of her doctoral studies; the guide was translated into English and Arabic versions and co-designed and piloted with the help of three Lebanese adults. MAK is Lebanese, speaks fluent English and Arabic and is deeply familiar with the cultural context of Lebanon.

Box 1. Interview guide

*Semi-structured Interview guide*

1. What is your understanding of obesity?
2. Are people in your community concerned about obesity?
3. Tell me about the opportunities that promote healthy eating that you have identified.
4. Tell me about the opportunities that promote physical activity that you have identified.
5. Tell me about the barriers to healthy eating that you have identified
6. Tell me about the barriers to physical activity that you have identified
7. Who are, in your opinion, the people or sectors that could contribute to promoting healthy eating behaviours and physical activity?
8. What actions could they do to help people in your community overcome the barriers to healthy eating behaviour and physical activity?

*Probes (examples)*

- Please discuss using the photographs that you have taken
- Please explain why you consider these as barriers/opportunities
- Would you give me an example?
- Can you give more details?

### Participants

The study was conducted in the Northern Governorate of Lebanon. The study inclusion criteria were Lebanese nationals, residents in North Lebanon, aged 20–64 years and owning and being able to use a camera. Exclusion criteria were individuals younger than 20 or older than 65 and those not owning or not able to use a camera. Purposive and convenience sampling strategies were used for recruitment, initiated through a community centre in Tripoli, which is visited by individuals from diverse demographic and socio-economic backgrounds. Snowball sampling was also employed, with participants invited to nominate others. To guide purposive sampling, MAK asked them to suggest individuals with varied demographics, including gender, age (20–35, 36–50, 51–64) and employment status (employed, unemployed, university student).

This approach identified thirty-six potential participants; twenty-five initially agreed to participate, of whom two withdrew for personal reasons, and three did not attend interviews. Therefore, a total of twenty adults participated in this study. Data saturation was reached after twenty interviews, with no new codes or themes emerging, so no further participants were recruited.

### Data collection

Individuals interested in participating in the study were provided with an information sheet detailing the study and the Photovoice process and offered two dates: one for an induction and another for an interview. In the first, written consent and demographic details were obtained; participants were also advised that participation was voluntary and that they could withdraw at any time from the study without consequence; the Photovoice process was outlined again, and the ‘mission’ (i.e. task) was provided: ‘*to photograph the opportunities and barriers that the participant observes that make it easier or harder for them to consume healthy food and to be physically active*’. The ‘mission’ and key terms (e.g. physical activity was defined as ‘*any movement done for leisure or transportation*’)^(21)^ and ethical considerations of the method (e.g. avoid *identifiable* photographs; avoid ‘anything’ causing harm) were also explained. Participants were instructed to take five to ten original photos per category in everyday settings (home, work, public spaces) and were given 2 weeks to complete the task. They were also advised that the documented barriers may not necessarily affect them personally but could reflect challenges others in their community face.

In the second meeting, participants brought selected images to guide face-to-face interviews conducted by the lead author (MAK) in a private space at the community centre in Tripoli. Participants did not receive any form of financial or material incentive for participating in the study to ensure voluntary participation. Participant recruitment and data collection were conducted between February 2018 and February 2019. Recruitment and data collection overlapped; interviews were scheduled based on each participant’s availability and completion of the Photovoice task.

### Data analysis

All interviews were audio recorded, transcribed verbatim and analysed in the interview language (English or Arabic). For interviews conducted in Arabic, E-Arabic was used for transcription, which is the Romanisation of the Arabic language^(22)^; this mainly depends on phoneme-to-grapheme writing, in other words, using Roman letters to write Arabic words as they sound^(22)^. Only extracted data (quotes) were translated into English. To increase the dependability of the data, randomly selected data (quotes) were back-translated by a Lebanese PhD colleague, and the original translation and the back translation were compared for accuracy^(23)^. Thematic analysis was used for analysis and interpretation to identify key themes and illustrate connections within the data^(24)^. While an inductive approach was intended, the socio-ecological model framed the overall study^(14)^, adding a deductive layer to the analysis. The principal researcher (MAK) read and re-read the transcripts, listened to the recordings and generated a list of codes. The ‘code-recode’ procedure was applied, where data were coded twice with a minimum of 14 days between each coding to enhance dependability^(25)^. Moreover, a selection of the transcribed and translated transcripts was read by the supervisors (LK) and (SF), and coding categories were discussed and negotiated in meetings involving all three authors to achieve a broad consensus on the coding scheme and shared insight into the phenomenon discussed; connections between codes were explored inductively and deductively using the socio-ecological model^(14)^. MAK’s cultural background as a Lebanese woman may have influenced her data interpretation. Nevertheless, ongoing reflexivity and collaborative discussions among the research team ensured a balanced perspective and strengthened the credibility of the findings.

## Results

A total of twenty Lebanese adults participated in the Photovoice study; the majority (*n* 9) were aged 20–35 years, *n* 6 were 36–50 years and *n* 5 were 51–64 years. Participant demographics are summarised in Table 1. Participants generated 257 photographs, with 118 and 139 representing negative and positive influences, respectively, and 20 interviews were conducted. The data from participants of different genders, age groups and occupational statuses provided a holistic understanding of how sociocultural, built and food environments influence behaviour. As such, findings were not compared across demographic groups, except in a few cases where notable differences emerged within the themes. Codes generated from the thematic analysis were initially integrated into seven themes (with sixteen subthemes): *(1) physical appearances and body concerns, (2) individual factors, (3) social influences, (4) media and communication, (5) food affordability and availability, (6) technology and leisure activities and (7) safety concerns*. However, as found in other contexts^(12,26,27)^, this study’s results show that Lebanon is no different in terms of individual factors (such as stress, lack of perceived available time to exercise, self-discipline, motivation) and unhealthy food advertisements on television and billboards that influence food choices and physical activity. Therefore, for this manuscript, we focused specifically on the six themes and ten subthemes that were unique to the Lebanese context, as they provided the most relevant insights for understanding Lebanon’s sociocultural and environmental factors.

**Table 1.** Study participants’ demographics

	Category	Frequency (*n*)
Total participants		20
Age group	20–35 years	9
	36–50 years	5
	51–64 years	6
Gender	Male	7
	Female	13
Occupation	Employed	10
	Unemployed	6
	Student/employed	1
	Student/unemployed	2
	Retired	1

### Theme 1: Perceptions towards obesity

All of the participants agreed in terms of the definition of obesity as excessive body fatness, with one noting, ‘*Obesity is being fat … being fat above the normal weight*’ (P14). However, discussions tended to focus more on body size and appearance than on the health implications of excess weight. Apparent differences also emerged between younger and older adults’ perceptions of a healthy or ideal body shape. In Lebanon, fuller bodies were traditionally associated with beauty, wealth and social status, and they were admired and valued. Paradoxically, as obesity rates have increased in Lebanon, slimmer body shapes are aggressively promoted and valued, while excessive body weight is shunned, resulting in negative attitudes, stigma and judgement directed at those who do not conform to the new body ‘ideal’ (Table 2).

**Table 2.** Quotes from theme 1, ‘Perceptions towards obesity’

Physical appearances and body concerns	*Quotes*
Perceptions towards obesity and body image	‘When I was your age [the researcher’s age – in the 20s], being fat was considered better than thin. We used to say that if you don’t have a large belly you’re not even worth a penny [a Lebanese saying], but it all changed now.’ (P20)
	‘You have a lot of social pressure in Lebanon to be thin, people are judgemental of your looks. It could be good sometimes. The good thing is that you always try to maintain your body weight.’ (P13)
	‘Obesity should be perceived as a health problem but here we consider it an appearances problem.’ (P4)
	‘After I discovered that I have a high cholesterol [blood cholesterol], I started to feel that I should change the way I eat and eat healthy.’ (P20)
	‘The Ministry of Public Health could do some awareness campaigns, that excess body weight causes health problems not only for the body. We should not see that our body is ugly and follow a diet. We should think about our health, that fatness causes heart problems.’ (P17)

Obesity is frequently seen as a health issue only when physical symptoms appear or when healthcare providers advise individuals to change their diet or lifestyle or lose weight. Some participants felt there should be less focus on appearance and more emphasis on recognising obesity as a medical condition with serious health risks, even when no visible illness is present (Table 2).

### Theme 2: Social circles

#### Eating together and social connections

Many highlighted the importance of social gatherings, dining out and cultural traditions such as meeting friends at coffee shops and smoking shisha as part of Lebanese culture. Some participants referred to shisha, a popular tobacco product, used in social settings that often involve prolonged sitting and a sedentary lifestyle. The majority felt that their social circles negatively influenced their food choices, although the opposite was also the case when their companions followed a healthy lifestyle. Practices like meal sharing, eating various meals, meze and buffets for celebrations are key features of Lebanese culture. However, regarding their health, the cultural focus on food, hospitality and food-sharing was perceived to encourage overeating. Discussions about the influence of family and social groups were mixed. Not all social influence was negative; for some participants, significant others were an essential source of motivation to change lifestyles, exercise and lose weight. Furthermore, having health concerns about a family member motivated other family members to lead healthier lifestyles and create a supportive home environment (Table 3).

**Table 3.** Quotations and pictures for theme 2, ‘Social influences’

*Social influences*	Quotation	Picture
*Eating out and eating together*	‘Here [referring to a picture] you can see shisha; I usually smoke shisha when I go to a coffee shop with my friends. We go a few times a week.’ (P16)	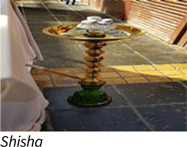
	‘We always order meals together to share. I can’t limit myself. I decide to order something healthy but when I see what they ordered I can’t say no and I end up eating with them.’ (P14)	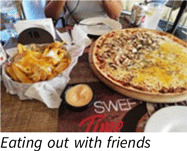
	‘As you could see in this picture you have a big variety of food, you end up eating a lot.’ (P7)	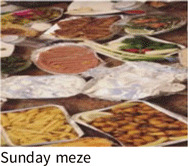
*Significant others and social connection*	‘I took a picture of this [a picture of oat bread] because we only eat oat bread at home because my dad discovered that he has cholesterol so our mom would only buy oat bread.’ (P2)	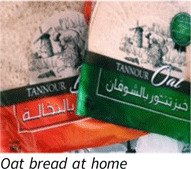
	*‘*Honestly, my sister and cousins motivate me to exercise. Now I joined fitness classes. For example, my sister was the first to go then my cousin wanted to go so all of us go now.’ (P17)	*No picture – information shared by the participant during the interview*
*The role of women*	*‘*You know there are still some parents who believe that the flavour of margarine [or ghee] is different. I know that margarine and oil contain the same number of calories, if you want to talk about calories. But it is not healthy. They say that they put a little. They usually use it for rice. I prefer that it gets cooked with oil but when the food is served for the family, it is not possible that I tell her [the mother] to cook for me alone, so sometimes I need to eat margarine.’ (P9)	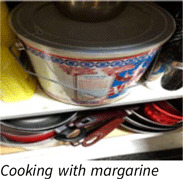
	‘In this picture you could see [Tabbouli – Lebanese salad] healthy food. It is available because my mom always tries to take the family towards a healthy lifestyle and she cooks meals that have a lot of vegetables, low margarine and oil.’ (P3)	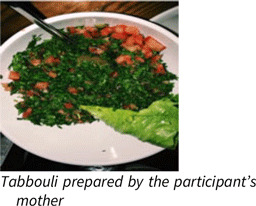

#### The role of women

In Lebanese culture, women are traditionally seen as the ones responsible for cooking for their families, a role that most participants felt positively influenced their family’s eating habits. Several participants perceived their mothers’ and wives’ cooking as an opportunity for healthy eating, describing their home-cooked meals as ‘healthy’. Some reported that they took homemade food to work, reducing the likelihood of buying fast food with colleagues. Additionally, women in Lebanon are mainly responsible for food procurement for the household and often set the rules or norms around controlling food availability at home (i.e. refusing to allow deliveries of fast food when they cook and insisting that the family eat home-cooked meals). A few participants, however, felt the opposite: that their mothers’ traditional cooking practices, such as using margarine or ghee, negatively influenced their food intake. Thus, it challenges the perception that traditional Lebanese dishes are always healthy. Women’s employment was perceived as a barrier to healthy eating for families, as it limits the time available for home cooking and increases reliance on convenient meals. Sometimes, someone else takes responsibility for food preparation, such as the woman’s mother, mother-in-law or the housekeeper, who may step in when there are working mothers. However, women are still likely responsible for managing the housekeeper’s cooking (Table 3).

### Theme 3: Media and communication

#### Media influencers and food bloggers

The media plays a dual role in influencing lifestyle behaviour among participants. Media outlets, television and social media were viewed positively for providing information on diet and physical activity. Moreover, some younger participants have reported that they get inspired by body transformation success stories of ‘bloggers’ and ‘influencers’ on social media, which motivates them to exercise. In contrast, other participants named a social media application and said that it negatively affects their eating behaviours; some ‘food bloggers’ that they follow share pictures of their meals while eating out and trying new restaurants, making them crave unhealthy meals. A participant explained that restaurants’ marketing strategies include sponsoring food bloggers invited to try the food for free or paid to share the pictures (Table 4).

**Table 4.** Quotations and pictures from theme 3, ‘Media and communication’

Subthemes	Quotation	Picture
*Social media influencers*	‘In my opinion, social media is playing a main role. When people see those influencers being fit and healthy and their bodies look good, this motivates the people our age to shift towards the healthy lifestyle, to work out and go to the gym.’ (P3)	*The picture was removed as it is a screenshot from the internet and contained the influencer’s body transformation pictures.*
	*‘*There are people on XXX [social media platform] who share things[information] that are very important advice and tips.’ (P11)	*The picture was removed as it contained the picture of a nutritionist on social media.*
*Food bloggers*	‘When you follow a page on XXX [social media platform] and they post a picture of those fries with cheddar cheese and those meals, the next day you want to go there.’ (P15)	*This picture was removed as it is a screenshot of a story posted by a food blogger on social media.*
	‘These pages belong to people who get to share their food on their page on XXX [social media platform] and sometimes are invited by the restaurants to try their food and of course to post some pictures.’ (P7)	*No picture – information shared by the participant during the interview*

### Theme 4: Food affordability and availability

#### Food availability and affordability

Regarding availability, smaller grocery stores and the settings where participants work or study often lack healthy food choices. In terms of affordability, more nutritious options were generally perceived as more expensive than alternatives with higher sugar, fat and calories. Although it was not an issue for all participants, many agreed that the economy in Lebanon has suffered considerably in recent years, and food prices have increased. Consequently, the affordability of healthy foods became an issue. Participants called for fiscal measures, reducing prices of healthy foods rather than imposing taxes on unhealthy ones, interventions by the government to stabilise food prices and influencing product formulation. Employed participants called for supportive work environments that encourage healthier lifestyles (including facilities such as fridges, microwaves and ovens) to reduce dependency on processed foods (Table 5).

**Table 5.** Quotations and pictures from theme 4, ‘Food affordability and availability’

	Quotation	Pictures
*Food availability and affordability*	‘This is a pack of toasts. When I saw it in the supermarket, I bought it right away – it contains 17 calories per slice, in the shops that are not that big you can’t find these things, you find the regular toast that contains 32 calories.’ (P15)	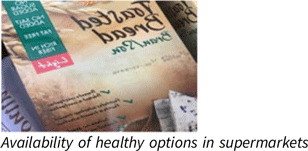
	‘This [referring to a picture], I remember that I went to buy healthy biscuits but I couldn’t find any.’ (P9)	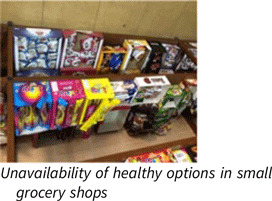
	‘When you go to the supermarket and you want to buy food, the healthy and sugar free ones are always more expensive than the regular ones. Who said that the underprivileged people don’t like to eat healthy? But when it is expensive, they would prefer to buy the cheaper product.’ (P3)	*The picture was removed as it includes sugar-free products [brand names are shown in this image]*
	*‘*The government should monitor the quality of the food, the ingredients they put in it. Healthier products should be cheaper so people would afford them.’ (P3)	*No picture – information shared by the participant during the interview*
*Fast food chains v*. *light menus and diet centres*	‘Now we have so many American restaurants XXX [fast food chains] all of them. XXX [referring to a picture] it is Lebanese, but it serves foreign food [burgers].’ (P18)	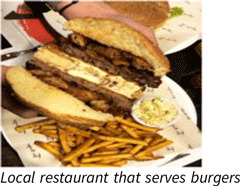
	‘Food boxes are meals, breakfast, lunch and dinner that get delivered to your home. You get to choose what to eat. They have a weekly menu so you will not think about what to eat during the day. No one cooks at home, so I find it the same if I cook or get these. Money wise, the unhealthy food is slightly cheaper but it will not be a big difference.’ (P13)	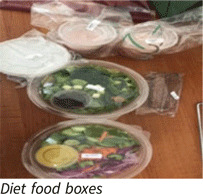
	‘The government should monitor the quality of the food. The ingredients they put in it. Healthier products should be cheaper so people would afford them.’ (P3)	*No picture – information shared by the participant during the interview*

#### Fast food chains *versus* light menus and diet centres

In the North Governorate, international fast-food chains and other local restaurants that serve ‘American food’, such as burgers and fried chicken, are widely available. In contrast, many participants said that traditional restaurants, which serve ‘light’ or ‘homemade’ traditional meals, are also widely available and provide opportunities for healthy eating. As one participant discussed, diet centres deliver ‘food boxes’, which include healthy meals tailored by nutritionists.

Although participants acknowledged that healthy choices are available in the form of traditional restaurants, they still stressed the need for the government to increase the availability and affordability of healthy options in workplaces and community settings (Table 5).

### Theme 5: Technology, modern lifestyle and physical activity environment

#### Modern technology and lifestyles

Modern technology, including cars, laptops and television subscription services for movies and video games, was widely photographed by participants as linked to increasingly sedentary lifestyles. One participant described television as her primary leisure activity because alternative leisure options are lacking in her community, especially during winter. Some described a noticeable shift in how Lebanese people use their leisure time, with children and young adults preferring video games and computers over traditional outdoor play. This was also associated with increased (unhealthy) snacking. Some mentioned smartphone and smartwatch applications as opportunities to increase physical activity and track caloric intake (Table 6).

**Table 6.** Quotations and pictures from theme 5, ‘Technology, modern lifestyle and physical activity environment’

Subtheme	Quotation	Picture
*Modern technology and lifestyles*	‘We don’t have anything to do here in Tripoli, we don’t have activities to do in the winter, we watch TV and while watching TV we feel like eating something. We order chips, chocolate, and nuts.’ (P12)	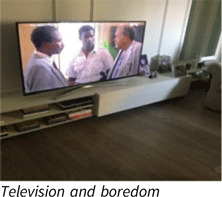
	‘All kids spend their time on their XXX [video game console] and these, you rarely see kids really playing.’ (P15)	*No picture – information shared by the participant during the interview*
	‘This is the health application on my phone. I always check how many steps I walked every day. It motivates me to walk more.’ (P10)	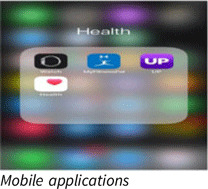
*Active hobbies and the accessibility to facilities*	‘All the things in Tripoli must be paid for. As in sports, for example, basketball, football and gyms all of these you have to pay to enter.’ (P3)	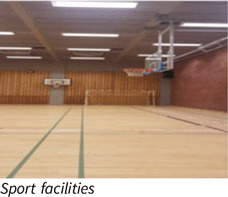
	‘This is the treadmill at the gym, the one that I am registered at. Of course, it is an opportunity for me that I can work out and maintain a healthy lifestyle – That’s why I took this picture.’ (P14)	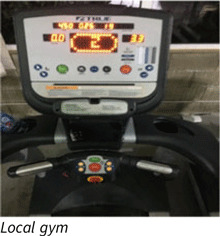
	I go on hikes. I really enjoy the views it’s very relaxing. Everyone can do the same. First because it’s free access – we have stunning views. Second, it’s everywhere, it’s safe, it’s not like walking on a treadmill. You are outdoors, you are doing physical activity and at the same time you are enjoying landscapes around you.’ (P13)	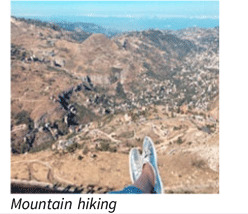
	‘In the summer I live in a beach resort, the good thing is that you have the chance to be more active [there] than to stay at home. The beach is in front of you, swimming is always a good idea. Second, we have a sandy shore, a very large one. They’ve put there a volleyball net, so you always have the option to play volleyball. If not, you can walk on the large sandy shore also we have courts for football, basketball. All of these help.’ (P9)	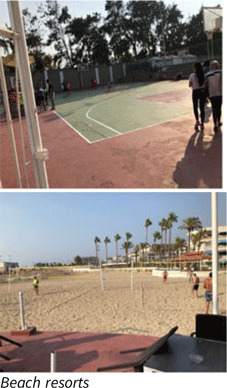

#### Active hobbies, community assets and accessibility to facilities

Several participants shared images of community and environmental assets such as the promenade (Corniche), the beach and natural surroundings (mountains) as opportunities for increasing outdoor activity. Additionally, many of the younger adults shared images of private gyms as their main hobby involving physical activity, providing the social and physical benefits of attending the gym. Similarly, images were shared by a small number of adults of private beach resorts offering facilities for active recreation such as swimming, playing volleyball on the beach, running, jogging or playing basketball or football. However, there is an inequality in terms of access to facilities. For many participants, gym memberships were not affordable, and the facilities were only accessible to private members. Participants called on the government to create more public facilities, offer subsidised gym memberships and organise community activities to encourage physical activity (Table 6).

### Theme 6: Safety concerns

Many female participants shared safety concerns impacting their outdoor physical activity engagement. Some women talked about the fear of receiving personal comments and the lack of security measures that inhibited them from walking in specific locations. Moreover, some cultural norms, for example, discouraging women from walking alone, further limit physical activity.

A couple of participants also cited a lack of traffic lights or pedestrian crossing lanes and the presence of motorcycles as barriers to walking. Moreover, several female participants explained that they would feel uncomfortable cycling on public roads to commute in Tripoli and prefer cycling in private or secured/gated areas (Table 7).

**Table 7.** Quotations and pictures from theme 6, ‘Safety concerns’

Subtheme	*Quotation*	Picture
*Women’s safety*	‘A lot of people [men] say comments … you don’t feel at ease while you are walking … so I prefer to go in my car and park in front of the store.’ (P2)	*No picture – information shared during the interview*
	‘My husband doesn’t like that my daughters walk alone ….’ (P19)	*No picture – information shared by the participant during the interview*
	‘Sometimes at night you feel there is not enough security … they say [government] that there is an increased security sometimes … but that’s only once per week … at night you don’t see police cars … roaming in the streets … I know a lot of countries that have police in the street at night even if there is nothing … just to make sure that it’s safe and secure ….’ (P9)	*No picture – information shared by the participant during the interview*
*Lack of good infrastructure*	‘You feel that in some streets … there is not a good pavement for people to walk … or when you are walking on a pavement you find a motorcycle or a bicycle … so when you’re walking you feel that that your hand is on your heart [Lebanese saying to express fear] that someone would bump into you.’ (P9)	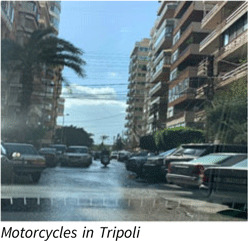

## Discussion

**Figure 1.**
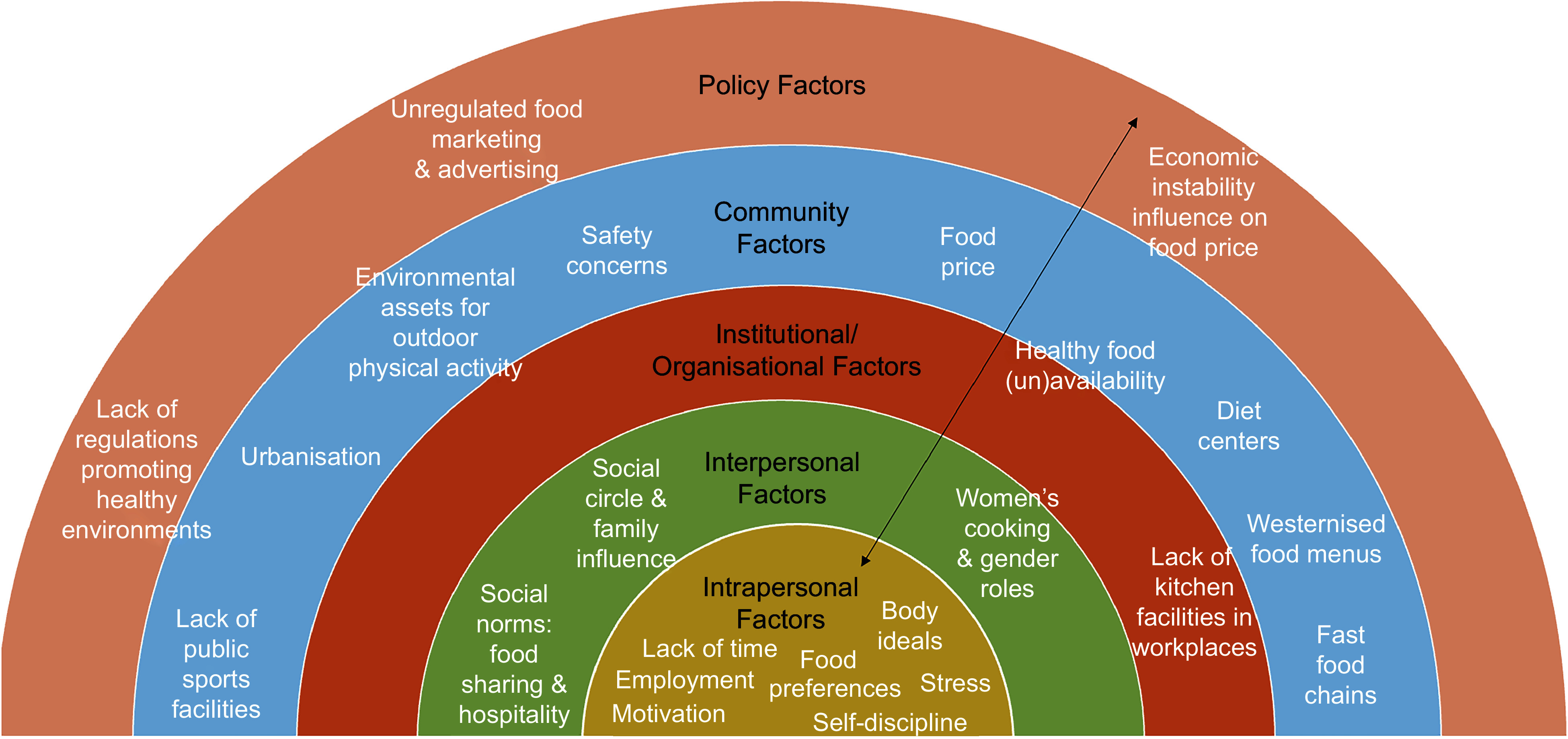
Socio-Ecological model of factors that influence adults’ eating behaviours and physical activity in North Lebanon (Adapted from McLeroy *et al.*, 1988).

This study contributes to the growing body of literature on the nutrition transition in Lebanon, highlighting shifts in dietary patterns, increased westernisation and sedentary activity^(4,5)^. It supports the well-documented shift from traditional diets to more Westernised eating patterns^(4,5)^, while also offering new qualitative perspectives on how Lebanese residents interpret and navigate these transitions. Participants shared photographs of viable options for eating out, many fast-food outlets and the availability of highly processed and fast foods. A noticeable shift towards sedentary lifestyles was also described to explain the increasing obesity in Lebanon. As reported elsewhere, children and young adults now rely heavily on electronic devices for work and leisure time, compared with traditional labour-intensive occupations and active outdoor play^(28)^. While this itself is not new, participants did, however, offer insight into how adults living in Lebanon view the rise in obesity occurring in society and several factors that may influence physical activity and healthy eating in this context. We used the five levels of the socio-ecological model^(14)^ together with prevention opportunities for policymakers to highlight the more salient themes. Figure 1 provides a summary of themes identified through the photovoice data, which are mapped directly into the socio-ecological model^(14)^ to illustrate the complex, interacting influences on behaviour.

### Intrapersonal Level

Our findings suggest that a paradox may exist in the views of adults living in Lebanon, where obesity is primarily viewed through the lens of body size and appearance, despite some recognition of its health risks. However, rates continue to rise quickly, occurring against a backdrop of shifting norms towards body size and the body ‘ideal’. Traditionally, in Arab countries, ‘plump’ bodies were highly valued as a symbol of beauty and wealth and thus desirable^(29)^. Food scarcity was recognised as shaping societal preferences for larger body sizes in developing countries^(30)^. The participants of this study discussed a shift towards Western cultural values of body image and thinness, with achieving a thinner body as a primary goal for the younger adults we interviewed. This shift could be linked to broader forces such as globalisation and urbanisation^(31)^. In addition, this drive for unrealistic and unachievable goals is potentially associated with increased body image dissatisfaction among young adults in the Arab region^(32)^. Future research could examine the factors driving these changing perceptions of body image in Lebanon, particularly focusing on how these shifts influence weight-related attitudes and behaviours, especially among youth.

Interestingly, while participants acknowledged the health risks associated with obesity, they often noted that it is only recognised as a health issue once physical symptoms or signs of illness appear. Moreover, many also noted that public and personal conversations tend to emphasise body size over health outcomes. For instance, a Lebanese cross-sectional study showed that people are generally aware of obesity’s links to CVD (80·27 %), hypertension (64·09 %) and certain cancers (48·56 %)^(28)^. While these figures suggest that many individuals recognise obesity health risks, our findings indicate that cultural factors such as a stronger focus on body image and appearance may overshadow these concerns in everyday conversations and decision-making.

### Interpersonal level

At the interpersonal level of the socio-ecological model, the focus is on understanding health-related behaviours and social interaction^(14)^. In the Eastern Mediterranean Region, people typically spend their leisure time with other people in communal spaces, and social gatherings are commonly preferred leisure activities^(33)^. In this regard, our findings show that leisure time was also spent mainly socialising with family or friends, commonly involving smoking shisha or sharing different meals or ‘meze’ at home or in restaurants, as food and hospitality are at the heart of Lebanese culture. Moreover, our study participants mainly perceived eating with others as linked to increased food consumption and unhealthy meals and encouraged overeating. However, positive social influences were also reported, such as adopting healthier habits modelled by close contacts, including joining exercise classes or consuming healthier meals. Some participants described social media influencers as sources of nutritional motivation or misinformation. These individual interactions with media and online personalities shaped their attitudes towards diet and exercise. As part of the Lebanese culture, supporting friends and family is regarded as ‘*the axis of Lebanese values, beliefs and culture*’^(34)^. In our study, caring about family members’ and significant others’ health concerns encouraged the participants to create a supportive environment to help them adopt healthier behaviours. Moreover, female family figures (mothers and wives) were found to play a vital role in influencing the eating behaviours of their families in terms of meal preparations as well as food procurement and setting the ‘rules’ at home (e.g. no food deliveries when food is cooked at home), which was viewed by many participants as positively impacting eating behaviours. While most perceive their mothers’ and wives’ traditional meals as healthy, few participants associate traditional Lebanese dishes with ‘unhealthy’ cooking practices, such as using margarine or ghee. Considerable research indicates the health benefits of traditional Lebanese diets^(1–3)^. The use of ghee or margarine as part of traditional cooking practices is an area that needs to be further explored.

Women’s (mothers’ and wives’) employment was perceived to hinder the participants’ eating behaviours as they could not cook for their families and needed to rely on convenient food and deliveries, except when women received support from their mothers or mothers-in-law or their ‘housekeepers’. Researchers have discussed that with economic advances and urbanisation, more women are in paid employment, and their roles are no longer limited to being housewives and cooking^(35,36)^. However, women’s responsibilities have not been replaced or shared by their spouses^(35)^. To ensure that daily meals are available for their family, they rely on more convenient meals cooked faster than traditional ones^(36)^. As identified in this study, patriarchy is culturally embedded in Lebanese society. While changing gender and social norms would be challenging and may take many years to achieve, social change may be promoted through campaigns and strategies that aim to strengthen the relationships between family members and distribute duties between them^(37)^.

### Institutional/organisational level

This level refers to formal and informal rules, policies and structures within organisations or institutions that influence health behaviours. The institutional environment includes food affordability, availability and accessibility of food options in various settings (e.g. home, school, work)^(13)^. As our participants explained, in some settings (e.g. workplaces and universities), lighter options with lower calories are not widely available and are more expensive than ‘unhealthy’ ones. This lack of healthier options reflects institutional-level barriers that may inadvertently promote unhealthy eating. A systematic review highlighted that food prices and limited access to shops selling healthy food are significant barriers to healthy eating for marginalised groups^(38)^.

In addition, the participants called for increased availability and affordability of ready-to-eat healthy food in work/university cafeterias. In this regard, settings-based interventions and national policies could be implemented to modify the quality of food sold in workplace cafeterias^(39)^. Also, as part of the Lebanese adults’ recommendations, the participants requested the inclusion of kitchen facilities (refrigerators and microwaves) at universities and worksites to support the consumption of traditional meals. Employers should recognise that obesity impacts businesses as it is associated with decreased productivity, increased healthcare claims and work-related injuries^(40)^. Therefore, companies are encouraged to integrate strategies that encourage employees to consume healthy food and be physically active into existing worksite health programmes^(41)^.

### Community level

At the community level, the findings show that international fast-food chains and other local restaurants that serve ‘American food’ are widely available in Tripoli. A study showed that the recipes of Lebanese dishes served in these restaurants were adapted based on Western dishes^(42)^. Local small grocery stores were also identified as part of the community food environment, where the availability and affordability of lighter or healthier food options are limited compared with less healthy alternatives. These restaurants, local food outlets and grocery shops constitute part of the broader community food environment, distinct from institutional food settings.

In addition to the food landscape, participants identified key physical and social features of the built environment that influence physical activity. Key environmental assets were identified, including the promenade (Corniche), beaches and natural surroundings, as opportunities for outdoor physical activity. However, safety is a key concern for people in North Lebanon; a couple of participants explained that unsafe environments, such as the lack of traffic lights or pedestrian crossing lanes and the presence of motorcycles, are barriers to walking. Moreover, as stated by some of our female participants, young female adults perceive their community social environment (e.g. hearing comments and cultural norms) and lack of safety as limiting their outdoor physical activity. The study participants have called for government municipalities and the police to introduce more safety measures and improve the built infrastructure to encourage physical activity. Evidence indicates that walking for leisure is linked to residents’ sense of safety in a neighbourhood^(43)^.

### Public policy level

At the broader public policy level, our participants suggested that the Lebanese government should raise awareness of the health consequences of obesity through media campaigns. However, they emphasised that these campaigns should be carefully designed to avoid reinforcing stigma. Evidence shows that mass media awareness campaigns could influence population knowledge and attitudes, yet evidence related to behaviour change is limited as these campaigns did not target the drivers of these behaviours^(44)^. To increase population knowledge and achieve meaningful and sustainable behaviour change, these awareness campaigns should be complemented with structural policies that specifically target cultural and economic drivers, including food marketing, body image norms and the affordability of nutritious food.

The participants supported subsidies to reduce the price of healthy/light products (e.g. low-fat and sugar-free products) to match the price of unhealthy ones (e.g. full-fat and sugar-sweetened products). Evidence shows that taxation and subsidies could reduce the price gap between healthy and unhealthy food and beverages. It can motivate people to choose nutritious options by decreasing the price gap between unhealthy and healthy products (e.g. sugar-sweetened beverages and milk) so they would make a choice that is less influenced by the product’s price^(45)^. To decrease the economic implications of these measures and to maximise their health benefits, tax revenues may also be used to support health promotion initiatives^(45)^. Future research could explore the feasibility of implementing such subsidies in Lebanon and evaluate their potential impact on dietary habits and cost-effectiveness.

In addition to food pricing, participants called for more support to promote physical activity at the national level. Policymakers might consider encouraging culturally appropriate messaging on increasing physical activity, including raising awareness of the benefits of outdoor pursuits and the opportunity to bond with friends and family to help increase active lifestyles in Lebanon. Many participants referred to the increased availability of private-access gyms, beach resorts and sports facilities that encourage physical activity. However, the high-cost fees restricted their use to those who could afford them and increased inequality in access to these facilities. As a solution, the participants advocated for an increased investment in public or low-cost recreational facilities, which has been associated with more active communities^(46)^.

Participants also raised concerns regarding the influence of unregulated food advertising, particularly those targeting younger audiences, as many described how advertisements for unhealthy foods, especially on social media, triggered cravings. Similar findings were observed in a study conducted in Iran, where television programmes that aim to increase nutrition knowledge were coupled with advertisements for unhealthy food^(47)^. Moreover, a randomised controlled trial conducted in Lebanon shows that exposure to ‘junk food content’ on social media was associated with increased hunger and cravings^(48)^. In the past, junk food advertisements were mainly seen on television; nowadays, the food industries target the youth by using social media and video games to advertise their products^(49)^. Policymakers are urged to collaborate with tech companies to develop guidelines that restrict unhealthy food advertisements targeting children and adolescents on social media^(50)^. Further research should evaluate the feasibility and effectiveness of such digital marketing regulations and related policy interventions.

### Strengths and limitations

A key strength of this study is the use of Photovoice, using photographs to guide interviews, which provides valuable insight into the environmental and cultural influences on individuals’ behaviours^(20)^. The participants were initially recruited from a community centre in Tripoli, and purposive and snowball sampling methods were employed to enable the inclusion of participants from diverse age groups, genders and employment status. However, the study has several limitations. First, it was conducted solely in the North Governorate, Lebanon, limiting its transferability to other regions. Further studies are needed to explore the perceptions of adults living in other Governorates in Lebanon. Second, the inclusion criterion requiring participants to own and be able to use a camera or smartphone with a camera may have unintentionally excluded individuals without access to such technology or with limited digital literacy. Although smartphone ownership was common among interested participants, future studies should consider providing equipment or technical support to ensure broader inclusivity. Moreover, participants’ health literacy was not assessed, though it could influence both their interview responses and the photographs they submitted. Therefore, it could be an important factor to consider in future research. Finally, this study did not collect data on participants’ weight status or BMI, as the focus was on general perceptions of obesity. Future studies should explore whether perspectives differ based on BMI, as individuals with higher BMI may have distinct experiences and views related to obesity.

### Conclusion

This study highlights the value of photovoice in capturing community perceptions of the factors contributing to obesity in Lebanon. Using the socio-ecological model, it illustrates how individual behaviours are shaped by cultural norms, social interactions and environmental and policy-level influences. These findings underscore the need for multi-level, context-specific strategies that go beyond awareness campaigns to address structural barriers and support healthier lifestyles. Future research should explore the applicability of such approaches across different regions and population groups in Lebanon.
